# Antenatal depressive symptoms and perinatal complications: a prospective study in rural Ethiopia

**DOI:** 10.1186/s12888-017-1462-4

**Published:** 2017-08-22

**Authors:** Tesera Bitew, Charlotte Hanlon, Eskinder Kebede, Simone Honikman, Abebaw Fekadu

**Affiliations:** 1 Department of Psychiatry, School of Medicine, College of Health Sciences, Addis Ababa University, Addis Ababa, Ethiopia; 2 Department of Psychology, Institute of Education and Behavioral Sciences, Debre Markos University, Debre Markos, Ethiopia; 3 Health Services and Population Research Department, King’s College London, Institute of Psychiatry, Psychology and Neuroscience, Centre for Global Mental Health, London, UK; 4 Department of Obstetrics and Gynecology, College of Health Sciences, Addis Ababa University, Addis Ababa, Ethiopia; 5 Department of Psychiatry and Mental Health, Perinatal Mental Health Project, Alan J Flisher Centre for Public Mental Health, University of Cape Town, Cape Town, South Africa; 6 Department of Psychological Medicine, Centre for Affective Disorders, King’s College London, Institute of Psychiatry, Psychology and Neuroscience, London, UK; 7 Center for Innovative Drug Development and Therapeutic Trials for Africa (CDT-Africa), Addis Ababa University, Addis Ababa, Ethiopia

**Keywords:** Antenatal depressive symptoms, Perinatal complications, Prospective study, Rural and low income, Ethiopia

## Abstract

**Background:**

Antenatal depressive symptoms affect around 12.3% of women in in low and middle income countries (LMICs) and data are accumulating about associations with adverse outcomes for mother and child. Studies from rural, low-income country community samples are limited. This paper aims to investigate whether antenatal depressive symptoms predict perinatal complications in a rural Ethiopia setting.

**Methods:**

A population-based prospective study was conducted in Sodo district, southern Ethiopia. A total of 1240 women recruited in the second and third trimesters of pregnancy were followed up until 4 to 12 weeks postpartum. Antenatal depressive symptoms were assessed using a locally validated version of the Patient Health Questionnaire (PHQ-9) that at a cut-off score of five or more indicates probable depression. Self-report of perinatal complications, categorised as maternal and neonatal were collected by using structured interviewer administered questionnaires at a median of eight weeks post-partum. Multivariate analysis was conducted to examine the association between antenatal depressive symptoms and self-reported perinatal complications.

**Result:**

A total of 28.7% of women had antenatal depressive symptoms (PHQ-9 score ≥ 5). Women with antenatal depressive symptoms had more than twice the odds of self-reported complications in pregnancy (OR=2.44, 95% CI: 1.84, 3.23), labour (OR= 1.84 95% CI: 1.34, 2.53) and the postpartum period (OR=1.70, 95% CI: 1.23, 2.35) compared to women without these symptoms. There was no association between antenatal depressive symptoms and pregnancy loss or neonatal death.

**Conclusion:**

Antenatal depressive symptoms are associated prospectively with self-reports of perinatal complications. Further research is necessary to further confirm these findings in a rural and poor context using objective measures of complications and investigating whether early detection and treatment of depressive symptoms reduces these complications.

## Background

Antenatal depression affects about 10% of women in High Income Countries (HICs) [1, 2], in Low and Middle Income Counties (LMICs) is higher, with some variation across studies. In these settings, the prevalence varies from 5% to nearly 40% [3–7] with a meta-analysis in 2004 of 17 studies from LMICs reporting a prevalence of 15.5% [2].

Antenatal depression is associated with increased functional impairment [8, 9], reduced self-care [10–12] and increased somatic complaints [9, 13, 14], which rise the risk of perinatal complications. Studies in HICs have reported association of antenatal depression with preeclampsia [15, 16], pregnancy and labor complications [17], premature contraction, increased use of analgesics and increased nausea during pregnancy [18]. Few studies from LMICs reported that antenatal depression was associated with increased risk of prolonged labor [19, 20], preeclampsia [20, 21] and increased risk of prolonged pregnancy [20].

There is accumulating evidence of adverse impacts of antenatal depression on the newborn [22, 23]. Low birth weight has been associated with antenatal depression in some [23, 24], but not all [19, 25], studies. No association between antenatal depression and neonatal mortality has been observed [20], but depression prior to pregnancy was associated with increased odds of stillbirth [26].

Most studies investigating the association between antenatal depression and perinatal complications were conducted in high income countries. Evidence about the impact on perinatal complications from rural community samples in LMICs is limited despite the high prevalence of both antenatal depression [5, 7] and perinatal complications [27–29] in such settings. Previous studies were limited by not controlling adequately for potential confounders, such as past history of adverse perinatal outcomes and chronic illnesses.

In our baseline study from a rural Ethiopian community, antenatal depressive symptoms were associated with increased non-scheduled antenatal care visits and pregnancy-related emergency visits [30], which indicates the possibility of an association of antenatal depressive symptoms with perinatal complications. The current study, therefore, aimed to investigate the effect of antenatal depressive symptoms on self-reported perinatal complications in rural Ethiopian women.

## Methods

### Study Design and setting

A population-based prospective study was conducted in Sodo district, southern Ethiopia, located approximately 100km from the capital city Addis Ababa. The district has a population of about 160,000 people and is divided into 58 sub-districts (‘*kebeles*’), four urban and 54 rural. Agriculture is the main source of income and the official language in the district is Amharic.

There are eight health centres (primary healthcare facilities) in the district and a health post for every sub-district. The health post is frontline primary healthcare facility staffed by community-based healthcare workers, Health Extension Workers (HEWs). HEWs are responsible for performing health prevention and promotion activities, to identify and monitor pregnant mothers and to maintain up-to-date maternal records in health posts. Members of health development army, a community-based network of health education volunteers each of whom covers five families, are also required to report pregnant women in their respective units to HEWs.

### Cohort Recruitment

The cohort was established by recruiting all consenting eligible pregnant women. These were women in their second and third trimesters of pregnancy, permanently residing in the study area at least for the preceding six months and without any cognitive and or hearing impairment that impaired adequate communication.

A network of community based healthcare workers (Health Extension Workers, HEWs), community based-healthcare education volunteers, and kebele chairmen and pregnant women themselves acted as key informants to identify all antenatal women in their respective sub-districts. The data collectors then conducted interview through home to home visits of identified women after informed consent had been obtained. Data collectors would declare potential participants “untraceable” after three recruiting visits had been unsuccessful.

A total of 1,355 women were identified within a three-month period, between early September and end of December, 2014. Of these, 44 identified antenatal women were in the first trimester of pregnancy and so were non-eligible [30]. Thus, a total of 1311 women were eligible and invited to participate. All eligible participants were prospectively followed up until 4-12 weeks (a median of eight weeks) after delivery. Four weeks was considered as an optimal time point to distinguish postpartum depression from postpartum blues as defined in DSM IV [31] and to reduce chance of recall bias.

### Sample size

The aforementioned sample size was estimated using EpiInfo version 7 [32] which was used to estimate the sample size for another paper [30] assuming statistical power of 80% and a 95% confidence interval. For this, a 19.9% prevalence of antenatal depressive symptoms [33] and a 34% antenatal care utilisation [34] as an outcome variable were used, assuming a 10% difference between women with and without antenatal depression.

### Data quality control and reporting

Forty experienced data collectors along with four supervisors conducted the data collection process after two days of training. All the data collectors were recruited from the local community and they had a minimum educational level of Grade 10. Most of them had either certificate or Diploma in relevant fields such as community healthcare. Among the supervisors, one had a Bachelor degree and the remaining had Diplomas in various related disciplines. The training was aimed to reduce respondent bias like social desirability bias by helping trainees understand the contents of the questionnaire, objectives and ethical issues relevant to the study. The data collection was closely monitored by the coordinator of the study through weekly meetings and regular telephone calls. Completed questionnaires were first checked by the supervisors, followed by the coordinator of the study and finally by the data entry clerks for consistency and missing data. Questionnaires deemed to have missing or inconsistent data were returned back to the data collectors for reinvestigation. Data were double entered using EpiData version 3.1 [32]. Data was reported in adherence to a checklist from STROBE statement [35] to maintain standard of reporting cohort data.

### Measurement

#### Outcome variables

The outcome variables were self-reported perinatal complications. The perinatal period is mostly defined as extending from 22 weeks of gestation to seven days after birth [36]. In this study, we used ‘perinatal complications’ to represent complications of the mother or the neonate that occurred between two weeks prior to childbirth and seven days after birth to reduce potential of recall bias. Perinatal complications were self-reported since both maternal healthcare services and maternal healthcare records in Ethiopia have major limitations [34, 37–39] and women have been shown to be more reliable source of such information [40].

Items adapted from the Ethiopian Demographic and Health Survey [34] were used to collect the data about potential perinatal complications. The instrument explored symptoms indicative of pregnancy complications two weeks prior to childbirth such as swollen hands/face, blurred vision, severe abdominal pain, discharge with unusual odor, pain during urination, severe headache, severe weakness. The following potential labour complications were explored: severe bleeding, severe headache, convulsions, high fever, loss of consciousness, labor lasting more than 12 hours, placenta not delivered within 30 minutes of the baby, tear, and premature rupture of membranes. Postpartum complications included: severe bleeding, blurred vision, convulsions, swollen hands and or face, high fever, malodorous vaginal discharge, loss of consciousness, severe headache, pain during urination, severe weakness, and difficulty of breathing and severe abdominal pain. Finally, complications affecting the neonate included: difficulty of breathing, yellow skin or eye color, poor sucking, pus or bleeding around umbilical cord, skin lesions or blisters, convulsions or rigidity, unconsciousness, red or swollen eyes with pus, any physical impairment and any physician diagnosed illness.

Adverse perinatal outcomes, including stillbirth, spontaneous abortion and neonatal mortality, were also assessed. Loss of pregnancy before 28 weeks of gestation was defined as “spontaneous abortion” and after 28 weeks “stillbirth” [41, 42]. Death of any live birth within 28 days was defined as “neonatal mortality”.

#### Primary exposure

For antenatal depressive symptoms, the primary exposure variable, assessment was made using a locally validated version of the Patient Health Questionnaire (PHQ-9) [43, 44] at a cut off of five or more indicating probable depression. Both the primary exposure and potential confounding variables were assessed at baseline, in the second trimester and the early third trimester [30].

#### Potential confounding variables

Potential confounders of the association between antenatal depression and perinatal complications, such as pregnancy intention, intimate partner violence, any chronic medical condition, receipt of care, life events and history of complications, were identified from the literature. Pregnancy intention was assessed using an item from Ethiopian Demographic Health Survey (EDHS) that asks whether mothers wanted to have the current pregnancy (“wanted”) or wanted to delay (“mistimed”) or never wanted it at all (“unwanted”) [34, 38]. Intimate partner violence was assessed using the Women’s Abuse Screening Test (WAST) with a five item scale [45]. Its score ranges 0-16 and a score greater than one indicates the presence of domestic violence [45].

The number of antenatal care visits was also recorded. The number of physician-diagnosed chronic medical conditions, including tuberculosis, HIV, renal disease and cardiac disease, were counted for each woman and recorded as “none” for those without any chronic medical conditions and “one or more” otherwise. Participants were also asked if they had a history of death of a child in the perinatal period, “history of adverse perinatal outcomes”. The number of threatening life events was assessed using a 12 item scale which has been adapted and used previously in Ethiopia [46]. Alcohol use was assessed using a four item scale, the Fast Alcohol Screening Test (FAST) [47]. The FAST score ranges from 0 to 16, where a score of three or more indicates hazardous or harmful drinking [47]. Socio-demographic and economic variables, including marital status, residence (rural/urban), monthly household income and level of education were also assessed. Household income was categorized into tertiles as “low”, “medium” and “high” income categories. Marital status was categorized as “married” or “single” since the number of unmarried, widowed and divorced women was small.

### Data analysis

Stata statistics software (version 13.1, Stata Corp, College Station, Texas) was used for data analysis. Spontaneous abortion and stillbirth were combined as “pregnancy loss” as the numbers were small. Two sample proportion test was used to explore whether baseline sample and follow-up samples differ in respect to selected baseline variables. Binary logistic regression was employed to compare the odds of pregnancy loss, neonatal mortality and experience of each of the self-reported perinatal complications for women with and without antenatal depressive symptoms. As an option to summarize the results and for ease of interpretation, the number of self-reported pregnancy complications were counted and dichotomized into “none” for those without any of the complications and “one or more” for those with one or more pregnancy complications. Similarly, the number of labour and postpartum complications was also dichotomized into “none” for those without any of respective complications and “one or more” for those with one or more respective complications. We combined these complications since maternal complications are often comorbid with one another [16, 17]. The total number of missing data for outcomes and loss to follow up was 71 (5.4%) including 30 women who didn’t deliver until end of follow up time. Thus, complete case analysis was used as it was suggested that less than 5% lost to follow up was with little concern [48, 49].

## Results

### Cohort characteristics

**Fig. 1:**
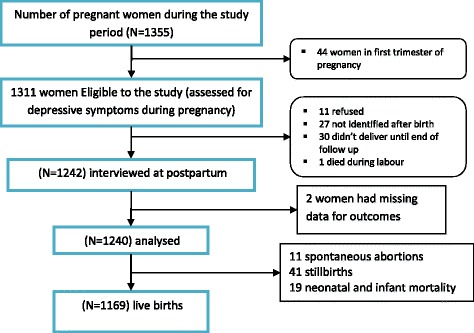
Diagram to show participant recruitment process

Out of 1311 pregnant women recruited into the study at baseline, 1240 (94.6%) were again interviewed at the follow-up time-point (median of eight weeks; interquartile range of 6-11 weeks postpartum) (Figure 1). Women in the complete follow-up sample were not significantly different in their baseline characteristics from the baseline sample. Of those followed-up at all-time points, nearly half of them (48%) were in the second trimester and the remaining were in the third trimester. More than one quarter of women (28.7%) had a PHQ-9 score of five or more indicating probable depression (20.9% had PHQ-9 score of 5-9 and 7.8% had PHQ-9 score of 10 or more) and 6.0% of them had reported hazardous level use of alcohol. Pregnancy was unintended in 43.8% of women, with 36.5% of pregnancies unwanted and 7.3% mistimed. The mean score of intimate partner violence was 2.1 (standard deviation (SD) of 2.9). The mean parity of participants was 2.7, SD = 2.1 (Table 1).

**Table 1:** Characteristics of participants

Characteristics		Baseline (*N*=1311)	Followed up (*N*=1240)	Pearson Chi^2^
Variables	Values	N (%)	N (%)	
PHQ-9 Status	PHQ < 5	924 (70.5)	884 (71.3)	0.2024 (*p* = 0.653)
	PHQ ≥ 5	387 (29.5)	356 (28.7)	
Marital Status	Married	1293 (98.6)	1225 (98.8)	0.1331 (*p* = 0.715)
	^a^Single	18 (1.4)	15 (1.2)	
Residence	Urban	103 (7.9)	98 (7.9)	0.0700 (*p* = 0.790)
	Rural	1208 (92.1)	1142 (92.1)	
Alcohol use	Non-users	875 (64.7)	828 (66.8)	0.0134 (*p*= 0.993)
	Mild level users	359 (27.4)	338 (27.3)	
	Harmful level users	77 (5.9)	74 (6.0)	
Household Income	High	429 (32.7)	414 (33.4)	0.1700 (*p* = 0.919)
	Medium	423 (32.3)	392 (31.6)	
	Low	459 (35.0)	434 (35.0)	
Mother's Education	Non-literate	878 (67.0)	841 (67.8)	0.2185 (*p* = 0.897)
	Grade 1-8	380 (29.0)	351 (28.3)	
	Grade 9-12 and above	53 (4.0)	48 (3.9)	
Parity	Nulliparous	254 (19.4)	226 (18.2)	0.5506 (*p* = 0.759)
	Primipara	193 (14.7)	185 (14.9)	
	Multipara	864 (65.9)	829 (66.8)	
Pregnancy intention	Wanted	734 (56.0)	696 (56.1)	0.1823 (*p* = 0.913)
	Mistimed	102 (7.8)	91 (7.3)	
	Unwanted	475 (36.2)	453 (36.5)	
Pregnancy complications	None	655 (50.1)	627 (50.6)	0.0926 (*p* = 0.761)
	One or more	656 (49.9)	613 (49.4)	
Chronic illness	None	871 (66.4)	832 (66.5)	0.0014 (*p*= 0.971)
	One or more	440 (33.6)	419 (33.5)	
Perinatal outcomes	Live birth	--	1169 (94.3)	--
	Pregnancy loss	--	52 (4.2)	
	Neonatal mortality		19 (1.5)	

^a^Single Marital Status= unmarried, widowed, divorced); Married = living with marital partner and married but living separate; Income was categorized into tertiles as low, medium and high; Social support score: minimum = 3; Maximum = 14; Mean=10.68; SD=2.00; Number of ANC visits: minimum = 0; Maximum= 8; Mean = 1.524; SD=1.50

### Antenatal depressive symptoms and potential perinatal complications

Scoring five or more on the PHQ-9 was associated with increased odds self-reports of pregnancy complications such as oedema [adjusted odds ratio (aOR = 2.68, 95% CI: 1.87, 3.83)], blurred vision (aOR = 1.89 95% CI: 1.41, 2.54), severe abdominal pain (aOR = 2.29, 95% CI: 1.73, 3.04), abnormal vaginal discharge (aOR = 2.08, 95% CI: 1.44, 2.98), burning sensation at urination (aOR = 1.67, 95% CI: 1.19, 2.34) and severe headache (aOR = 1.96, 95% CI: 1.49, 2.58) (Table 2). There was a more than two times increased odd of one or more (composite) pregnancy complications among women with antenatal depressive symptoms (aOR = 2.44, 95% CI: 1.84, 3.23).

**Table 2:** Impact of having antenatal depressive symptoms on each of self-reported perinatal complications

Self-Reported Perinatal Complications	During Pregnancy		During Labour		Up to 7 days Postpartum		Neonatal	
	n (%)	aOR (95% CI)	n (%)	aOR (95% CI)	n (%)	aOR (95% CI)	n (%)	aOR (95% CI)
Edema	172 (13.8)	2.68 (1.87, 3.83)		--	70 (5.6)	2.37 (1.39, 4.04)	--	--
Blurred vision	293 (23.6)	1.89 (1.41, 2.54)		--	301(24.3)	1.74 (1.29, 2.36)	--	--
Severe abdominal pain	349 (28.2)	2.29 (1.73, 3.04)		--	382 (30.8)	1.61(1.21, 2.15)	--	--
Abnormal discharge	163 (13.2)	2.08 (1.44, 2.98)		--	187 (15.1)	1.42 (0.99, 2.02)	--	--
Burning sensation at urination	201 (16.2)	1.67 (1.19, 2.34)		--	257 (20.7)	1.59 (1.16, 2.18)	--	--
Severe headache	398 (32.1)	1.96 (1.49, 2.58)	340 (27.4)	1.84 (1.37, 2.46)	338 (27.3)	1.68 (1.24, 2.25)	--	--
Convulsion	--	--	491 (39.6)	1.42 (1.07, 1.87)	257 (20.7)	1.69 (1.24, 2.32)	--	--
Haemorrhage	--	--	378 (30.5)	1.74 (1.31, 2.33)	420 (33.8)	1.60 (1.22, 2.17)	--	--
Unconsciousness	--	--	152 (12.3)	1.48 (1.00, 2.19)	41 (3.3)	2.45 (1.25, 4.80)	--	--
Fever	--	--	368 (29.8)	1.98 (1.48, 2.64)	274 (22.1)	1.87 (1.37, 2.54)	--	--
Premature Rupture of Membrane	--	--	92 (7.4)	1.81 (1.11, 2.92)		--	--	--
Prolonged labour	--	--	264 (21.3)	1.98 (1.43, 2.73)		--	--	--
Tear	--	--	92 (7.4)	1.19 (0.72, 1.99)		--	--	--
Retained placenta (30 min)	--	--	66 (13.4)	1.57 (1.08, 2.30)		--	--	--
Difficulty or Fast Breathing	--	--	--	--	--	--	129 (11.0)	1.70 (1.12, 2.58)
Yellow Skin/Eye Color (Jaundice)	--	--	--	--	--	--	45 (3.8)	1.08 (0.53, 2.19)
Poor Sucking or Feeding	--	--	--	--	--	--	48 (4.1)	0.76 (0.39, 1.50)
Pus, Bleeding around Umbilical Cord	--	--	--	--	--	--	162 (13.8)	1.38 (0.94, 2.01)
Skin Lesions Or Blisters	--	--	--	--	--	--	129 (10.1)	1.18 (0.77, 1.80)
Convulsions/Spasms/Rigidity	--	--	--	--	--	--	115 (9.8)	1.72 (1.12, 2.65)
Lethargy/Unconsciousness	--	--	--	--	--	--	35 (3.0)	1.11 (0.51, 2.39)
Red or Swollen Eyes With Pus	--	--	--	--	--	--	53 (4.5)	1.50 (0.80, 2.80)
One or more of any symptoms	700 (56.4)	2.44 (1.84, 3.23)	826 (66.6)	1.84 (1.34, 2.53)	804 (64.8)	1.70 (1.23, 2.35)	418 (35.6)	1.30 (0.98, 1.73)

N = total sample size; n = number of cases having perinatal complications ( % = percentage of cases having perinatal complications)

Controlled for alcohol use, residence, marital status, household income, education, intimate partner violence, life threatening events, number of ANC visits, parity, pregnancy intention, chronic conditions, and previous experience of adverse perinatal outcomes in all models and after adjusting for presence of one or more of any pregnancy complications symptoms and delivery complications in modelling each of labour complications and postpartum complication respectively.

Women with antenatal depressive symptoms had increased odds of labour complications [severe headache (aOR=1.84, 95% CI: 1.37, 2.46), convulsion (aOR = 1.42, 95% CI: 1.07, 1.87), haemorrhage (aOR = 1.74, 95% CI: 1.31, 2.33), unconsciousness (aOR = 1.48, 95% CI: 1.00, 2.19), fever (aOR = 1.98, 95% CI: 1.48, 2.64), premature rupture of membranes (aOR = 1.81, 95% CI: 1.11, 2.92), prolonged labour (aOR = 1.98, 95% CI: 1.43, 2.73) and retained placenta (aOR = 1.57, 95% CI: 1.08, 2.30)] (Table 2). Women with antenatal depressive symptoms also had increased odds of one or more (composite) labour complications (aOR = 1.84, 95% CI: 1.34, 2.53).

Among women with antenatal depressive symptoms, there was increased odds of all postpartum complications [edema (aOR=2.37, 95% CI: 1.39, 4.04), blurred vision (aOR=1.74, 95% CI: 1.29, 2.36), severe abdominal pain (aOR=1.61, 95% CI: 1.21, 2.15), burning sensation at urination (aOR=1.59, 95% CI: 1.16, 2.18), severe headache (aOR=1.68, 95% CI: 1.24, 2.25), convulsion (aOR=1.69, 95% CI: 1.24, 2.32), haemorrhage (aOR=1.60, 95% CI: 1.22, 2.17), unconsciousness (aOR=2.45, 95% CI: 1.25, 4.80) and fever (aOR=1.87, 95% CI: 1.37, 2.54)] (Table 2). Women with antenatal depressive symptoms also had increased odds of composite postpartum complications (aOR=1.70, 95% CI: 1.23, 2.35) in the multivariate model (Table 2).

In the multivariate model (Table 2), neonates of women with depressive symptoms had increased odds of difficulty of breathing or fast breathing (aOR = 1.70, 95% CI: 1.12, 2.58) and convulsions or spasms (aOR = 1.72, 95% CI: 1.12, 2.65) compared to women without depressive symptoms according to women’s self-reported measures. But, the association of antenatal depressive symptoms with composite neonatal complications became marginally non-significant in the multivariate model (OR=1.30, 95% CI: 0.98, 1.73) (Table 2). However, when severity of antenatal depressive symptoms was considered, the odds of each of the potential perinatal complications (maternal and neonatal) did not consistently increase with severity (Table 3). In the multivariate model, antenatal depressive symptoms were not associated with pregnancy loss (aOR = 1.26, 95% CI: 0.65, 2.44) or neonatal mortality (aOR = 2.03, 95% CI: 0.73, 5.63) (Table 4).

**Table 3:** Impact of Antenatal depressive symptoms on each of perinatal complications

	Potential perinatal complications	OR (95% CI)		
		Mild Depression	Major depression	Risk/protective factors
Pregnancy complications	Edema	2.86 (1.95, 4.17)	2.15 (1.19, 3.88)	^a^Mild Alcohol use
	Blurred vision	2.11 (1.54, 2.91)	1.33 (0.80, 2.20)	^a^(IPV, LTET, history perinatal mortality)
	Severe headache	1.81 (1.34, 2.45)	2.47 (1.57, 3.90)	^a^(Primary schooling, increased LTET)
	Severe abdominal pain	2.41 (1.78, 3.27)	1.96 (1.23, 3.13)	
	Abnormal discharge	2.17 (1.48, 3.20)	1.78 (0.98, 3.24)	^b^High income
	Burning sensation at urination	1.50 (1.04, 2.18)	2.24 (1.32, 3.79)	
	One or more of any symptoms	2.47 (1.81, 3.38)	2.34 (1.44, 3.82)	^b^(ANC use and high income)
labour complications	Haemorrhage	1.94 (1.42, 2.64)	1.22 (0.75, 1.98)	^b^(secondary schooling and more)
	Convulsion	1.49 (1.10, 2.01)	1.21 (0.76, 1.91)	^b^higher parity, †pregnancy complications
	Severe headache	1.87 (1.36, 2.57)	1.72 (1.07, 2.78)	^b^primary schooling, †pregnancy comps.
	Unconsciousness	1.51 (0.99, 2.29)	1.39 (0.74, 2.61)	^a^pregnancy complications
	Fever	2.01 (1.47, 2.74)	1.88 (1.17, 2.99)	^b^Primary schooling, ×parity, †preg. comps
	Premature Rupture of Membrane	2.00 (1.21, 3.31)	1.26 (0.56, 2.83)	^a^having comorbid conditions
	Prolonged labour	2.10 (1.48, 2.96)	1.62 (0.95, 2.76)	^a^ANC Use, ×parity, †pregnancy comps.
	Tear	1.01 (0.56, 1.83)	1.72 (0.82, 3.61)	^a^Mild Alcohol use, ×parity, †preg. comps.
	Retained placenta (30 min)	1.66 (1.11, 2.49)	1.29 (0.69, 2.42)	^a^pregnancy comps.
	One or more of any symptoms	2.12 (1.48, 3.04)	1.20 (0.71, 2.01)	^b^parity, †pregnancy comps.
postnatal complications	Haemorrhage	1.51 (1.11, 2.06)	1.98 (1.23, 3.20)	^b^Rural, †medium income, †primary schooling, †unwanted pregnancy, †history perinatal mortality
	Edema	2.18 (1.23, 3.87)	3.03 (1.36, 6.75)	^a^Intimate partner violence, †labour comps.
	Blurred vision	1.72 (1.24, 2.39)	1.87 (1.15, 3.04)	^b^Rural, ×increased income and education, †mistimed pregnancy, †labour comps.
	Convulsion	1.71 (1.22, 2.41)	1.59 (0.94, 2.68)	^b^Rural, ≥secondary schooling, †labour comp.
	Severe headache	1.40 (1.01, 1.94)	2.95 (1.81, 4.78)	^b^Prim schooling, †increased LTET, †labour comps
	Unconsciousness	2.27 (1.10, 4.70)	2.98 (1.16, 7.64)	^a^increased LTET, †labour complications
	Severe abdominal pain	1.54 (1.13, 2.11)	1.87 (1.16, 3.01)	^b^Alcohol use, ×high income, †labour comps
	Abnormal discharge	1.35 (0.92, 1.99)	1.59 (0.90, 2.80)	^a^labour comps
	Burning sensation at urination	1.20 (0.84, 1.71)	3.20 (1.97, 5.19)	^b^higher income, †secondary schooling, †labour comps
	Fever	1.74 (1.24, 2.43)	2.45 (1.49, 4.02)	Incm3-, increased labour comps
	One or more of any symptoms	1.33 (0.94, 1.88)	4.16 (2.11, 8.20)	^b^Mild alcohol use, ×high income and education, ×use of ANC, †labour comps
neonatal complications	Difficulty or Fast Breathing	1.67 (1.07, 2.60)	2.19 (1.18, 4.06)	^a^Intimate partner violence
	Yellow Skin/Eye Color (Jaundice)	1.34 (0.64, 2.79)	0.95 (0.30, 3.05)	^b^Increased parity, †unwanted pregnancy, † history perinatal mortality
	Poor Sucking or Feeding	0.72 (0.32, 1.61)	1.38 (0.54, 3.53)	^a^Mild alcohol use
	Pus, Bleeding, around Umbilical Cord	1.61 (1.08, 2.40)	1.66 (0.91, 3.01)	^b^High income
	Skin Lesions Or Blisters	1.38 (0.89, 2.15)	1.08 (0.53, 2.20)	^a^Comorbid conditions
	Convulsions/Spasms/Rigidity	2.08 (1.33, 3.26)	1.79 (0.90, 3.53)	^a^ history perinatal mortality
	Lethargy/Unconsciousness	1.19 (0.51, 2.78)	1.75 (0.59, 5.19)	
	Red or Swollen Eyes With Pus	1.54 (1.14, 2.07)	1.52 (0.95, 2.43)	^b^High income
	Difficulty or Fast Breathing	1.67 (1.07, 2.60)	2.19 (1.18, 4.06)	^a^intimate partner violence

Reference group: women with minimal depression; ^a^ = risk factor; ^b^ = protective factor

After controlling for alcohol use, residence, marital status, family income, educational level, intimate partner violence (IPV), life threatening events (LTET), parity, pregnancy intention, previous history of adverse events, pregnancy complications and chronic medical conditions.

**Table 4:** Antenatal Depressive symptoms vs adverse perinatal outcomes

Characteristics	Pregnancy loss		Neonatal mortality		Either pregnancy loss or neonatal mortality	
	(cOR, 95% CI)	(aOR, 95% CI)	(cOR, 95% CI)	(aOR, 95% CI)	(cOR, 95% CI)	(aOR, 95% CI)
Depressive Symptoms: PHQ9≥5	1.12 (0.61, 2.05)	1.26 (0.65, 2.44)	1.83 ( 0.73, 4.60)	2.03 (0.73, 5.63)	1.29 (0.77, 2.14)	1.44 (0.82, 2.53)
Residence: Rural	1.44 (0.44, 4.72)	2.25 (0.44, 11.42)	omitted	omitted	2.00 (0.62, 6.49)	2.84 (0.60, 13.22)
House hold Income: High	0.94 (0.49, 1.81)	0.84 (0.42, 1.70)	1.22 ( 0.41, 3.67)	1.43 (0.45, 4.56)	1.01 (0.57, 1.78)	0.97 (0.53, 1.79)
Medium	0.77 (0.38, 1.54)	0.72 (0.35, 1.47)	1.10 (0.35, 3.43)	1.20 (0.38, 3.82)	0.84 (0.46, 1.54)	0.83 (0.45, 1.54)
Low	1	1	1	1	1	1
Educational Level: Secondary +	1.51 (0.45, 5.09)	2.66 (0.46, 15.24)	omitted	1.00	1.08 (0.32, 3.59)	2.21 (0.53, 1.88)
Primary Schooling	0.95 (0.51, 1.80)	1.02 (0.49, 2.11)	0.85 (0.30, 2.38)	0.99 (0.31, 3.18)	0.92 (0.54, 1.59)	1.01 (0.42, 11.40)
Non-literate	1	1	1	1	1	1
Intimate Partner violence	0.85 (0.74, 0.98)	0.85 (0.73, 0.98)	0.98 ( 0.83, 1.15)	0.99 (0.82, 1.18)	0.89 (0.80, 0.99)	0.89 (0.79, 1.00)
Threatening life events	0.84 (0.65, 1.08)	0.86 (0.65, 1.14)	0.87 ( 0.58, 1.30)	0.82 (0.53, 1.29)	0.84 (0.68, 1.05)	0.84 (0.66, 1.08)
Parity	0.98 (0.86, 1.12)	1.01 (0.85, 1.19)	1.07 ( 0.87, 1.32)	1.03 (0.80, 1.33)	1.00 (0.90, 1.13)	1.01 (0.88, 1.17)
Pregnancy Intention: Wanted	1	1	1	1	1	1
Mistimed	0.89( 0.31, 2.57)	1.00 (0.34, 2.96)	0.63 (0.08, 4.90)	0.55 (0.07, 4.41)	0.82 (0.32, 2.12)	0.86 (0.32, 2.27)
Unwanted	0.62 (0.33, 1.17)	0.61 (0.31, 1.20	0.75 ( 0.28, 2.01)	0.61 (0.22, 1.73)	0.65 (0.38, 1.12)	0.61 (0.34, 1.09)
History of adverse perinatal outcomes	1.48 (0.82, 2.66**)**	1.66 (0.89, 3.11)	1.63 ( 0.64, 4.18)	1.47 (0.54, 3.98)	1.52 (0.92, 2.51)	1.61 (0.94, 2.76)
pregnancy complications	1.21 (0.69, 2.10)	1.37 (0.74, 2.54)	1.15 ( 0.46, 2.85)	1.00 (0.45, 2.23)	1.19 (0.74, 1.92)	1.28 (0.75, 2.17)
other comorbid illness	0.91 (0.53, 1.54)	0.89 (0.51, 1.55)	1.12 ( 0.52, 2.42)	1.07 (0.39, 2.90)	-	0.93 (0.58, 1.48)

## Discussion

Antenatal depressive symptoms and self-reported perinatal complications were common in the study area. More than half of participants reported having at least one or more of self-reported perinatal complications. In similar non-clinical studies, which reported patient defined perinatal complications, almost 50% of the participants reported some type of illness during pregnancy [50]. Similarly, a study of non-severe maternal morbidity in Malawi and Pakistan reported that 50.1% and 53% of women respectively reported having at least one pregnancy complications, infective or non-infective [29].

Depressive symptoms found to be associated with up-to three times increased odds of almost all self-reported symptoms of perinatal complications during pregnancy, delivery and postpartum. The associations of antenatal depressive symptoms with pregnancy loss and neonatal mortality were non-significant. The study found consistently strong association of antenatal depressive symptoms with all self-reports of perinatal complications in LMICs settings where there is lack objective evidence about perinatal complications. The finding has future importance in designing intervention strategies of antenatal depressive symptoms so as to improve the risk of perinatal complications.

The increased odds of self-reports of perinatal complications among women with antenatal depressive symptoms in our study supports studies in HICs where antenatal depression was associated with a pre-eclampsia [15, 16], wide range of other individual and combined pregnancy complications such as gestational hypertension, premature rupture of membranes, and various infections (Urinary Tract Infections, cervical, vaginal, intra-amniotic infections) [16, 17]. Our study also supports increased association of antenatal depressive symptoms with individual and combined delivery complications (assisted delivery, non-progressive labor, shoulder dystocia, postpartum hemorrhage, meconium, and suspected sepsis) [16], premature contraction, use of analgesics and increased nausea during pregnancy [18].

Few existing studies in LMICs also demonstrated that antenatal depression was associated with increased risk of prolonged labor [19, 20, 51], assisted deliveries, labor and delivery complications such as vaginal tear, unconsciousness, heavy vaginal bleeding during delivery and at postpartum, fever, malodorous vaginal discharge and leaking urine or faeces [51] as well as preeclampsia [20, 21].

The strong association of antenatal depressive symptoms with self-reports of perinatal complications may be explained by changes in life style [52], increased disability [8, 9], malnutrition [53] and food insecurity [54], reduced social support [55] and self-care [10–12] among women with antenatal depressive symptoms that delay timely healthcare seeking resulting in worsened perinatal complications among these group of women. Thus, depression may either worsen existing complications or increase vulnerability of women for developing complications (infection, hypertension, unsafe abortion) by reducing women’s self-care, social support and functioning. But evidence regarding the impact of depression on perinatal complications through biochemical changes is mixed [56].

Although we did not enquire about pre-eclampsia specifically, the significantly higher rate of symptoms like oedema, blurred vision and convulsions around two weeks before birth among women with antenatal depressive symptoms suggests an elevated risk of pre-eclampsia, which is borne out in the literature from other settings [15, 16, 20, 21]. The findings of the increased odds of symptoms of fever and pain during urination and abdominal pain also suggest the risk of infection may be increased among women with antenatal depressive symptoms [16, 57]. It can also be linked to increased effect of depression on pelvic inflammatory diseases reported in the general population [58].

Our finding about the increased odds of prolonged labour among women with depressive symptoms replicated the findings of cohort studies in Ghana [51], Ethiopia [19] and China [20]. It also supports the increased risk of shoulder dystocia and assisted delivery reported in HICs [16]. The association between antenatal depressive symptoms and prolonged labor in LMIC settings may be explained partly by reduced self-efficacy to push during labor due to fear of childbirth in a setting where there is increased maternal mortality. It may also be explained by malnutrition that may disable normal progression of labor. Increased odds of intra-partum and postpartum haemorrhage and unconsciousness in our study also accorded with the findings in Ghana that reported increased loss of consciousness and heavy vaginal bleeding at and after birth among women with antenatal depression [51].

Our finding of strong association between antenatal depressive symptoms and perinatal complications may also be explained by somatic symptoms associated with depression. These somatic symptoms are highly prevalent among people with mild and moderate depression group compared to people with severe depression group (a group characterized by motor retardation than somatization) [59]. Mild depression, compared to major depression, is again higher among pregnant women than non-pregnant women [60] implying higher prevalence of somatic symptoms among these group of women which could be presented as perinatal complications.

Our finding of an increased odds of potential neonatal complication also accords with findings in other settings of increased risk of both severe neonatal illness [51] and risk of fetal distress [17] among women with antenatal depression. But, our study didn’t support the non-significant odds of fetal distress reported in China [20].

The non-significant finding of the association between antenatal depressive symptoms and adverse perinatal outcomes in our study may be due to low statistical power. Nevertheless, a large sample size cohort study in Ghana has also reported non-significant association of PHQ-9 assessed antenatal depressive symptoms with stillbirth and neonatal mortality [51]. Similarly another study in UK [26] that distinguished current antenatal depression, from pre-existing depression prior to pregnancy, demonstrated that current antenatal depression was not a predictor of stillbirth. The other studies [17, 19, 20] also reported non-significant findings though they too may have been limited by low statistical power. Nevertheless, our study did not distinguish current antenatal depressive symptoms from prior-pregnancy depressive symptoms. However, the current study was part of a bigger project aimed to initiate integration of mental healthcare in primary healthcare facility. Thus, prior to the commencement of the study, there was no accessible mental healthcare service in the area for women to get medical prescriptions or other healthcare interventions for mental health problems.

The strength of our study was it was population based prospective design with greater chance of generalizability to all perinatal women. Our use of a locally validated measure of antenatal depressive symptoms increased validity of exposure. However, the relatively strong association of the antenatal depressive symptoms with potential perinatal complications in our study may be explained by greater levels of somatic symptoms among these women, attrition bias and the overlap of normal symptoms of pregnancy with depressive symptoms. However, we have previously noted for this population that somatic symptoms, as a presentation of perinatal depression relates to disability and morbidity [9]. There is consistent evidence about the association of depression and somatisation, with somatization being an important manifestation of depression [13]. These two disorders co-occur together and were termed as ‘common mental disorders’ [5, 9, 13, 14, 19, 61, 62]. The association between antenatal depressive symptoms and self-reported perinatal complications in the postnatal period may be due to negative symptom recall bias among women with postnatal depressive symptoms. However, it is important to note that about 60% of maternal mortality occurs during the postpartum, indicating increased complications within this period [63] that are associated with antenatal depressive symptoms persisting to postpartum.

With the poor quality health record system in Ethiopia [34, 37–39], we were required to rely on self-report of the participants to document perinatal complications. This self-report may reflect either under or over-reporting of complication types, severity or timing of outcome events. But, the measures had potential to assess perceived ill health of the participants, a neglected area of healthcare despite currently promoted patient-centred healthcare philosophy in the study area and around the world. Data collectors would declare potential participants “untraceable” after three recruiting visits had been unsuccessful. It is possible that these participants were ‘untraceable’ due to their being depressed and or due to physical health problems.

## Conclusion

Antenatal depressive symptoms appear to have considerable effect on self-reported perinatal complications. Further studies should look at whether early detection and treatment of antenatal depressive symptoms would reduce the risk of perinatal complications.

## Abbreviations

aOR: adjusted Odds Ratio
CI: Confidence Interval
cOR: crude Odds Ratio
EPDS: Edinburgh Postnatal Depression Scale
HICs: High Income Countries
LBW: Low Birth Weight
LMIC: Low and Middle Income Countries
PHQ-9: Patient Health Questionnaire-9
SRQ: Self Reporting Questionnaire
UK: United Kingdom
USA: United States of America
